# Genome-wide association study reveals white lupin candidate gene involved in anthracnose resistance

**DOI:** 10.1007/s00122-021-04014-7

**Published:** 2022-01-05

**Authors:** Joris A. Alkemade, Nelson Nazzicari, Monika M. Messmer, Paolo Annicchiarico, Barbara Ferrari, Ralf T. Voegele, Maria R. Finckh, Christine Arncken, Pierre Hohmann

**Affiliations:** 1 Department of Crop Sciences, Research Institute of Organic Agriculture (FiBL), Frick, Switzerland; 2 Research Centre for Animal Production and Aquaculture, CREA, Lodi, Italy; 3 Institute of Phytomedicine, University of Hohenheim, Stuttgart, Germany; 4 Department of Ecological Plant Protection, University of Kassel, Witzenhausen, Germany

## Abstract

**Key message:**

GWAS identifies candidate gene controlling resistance to anthracnose disease in white lupin.

**Abstract:**

White lupin (*Lupinus albus* L.) is a promising grain legume to meet the growing demand for plant-based protein. Its cultivation, however, is severely threatened by anthracnose disease caused by the fungal pathogen *Colletotrichum lupini*. To dissect the genetic architecture for anthracnose resistance, genotyping by sequencing was performed on white lupin accessions collected from the center of domestication and traditional cultivation regions. GBS resulted in 4611 high-quality single-nucleotide polymorphisms (SNPs) for 181 accessions, which were combined with resistance data observed under controlled conditions to perform a genome-wide association study (GWAS). Obtained disease phenotypes were shown to highly correlate with overall three-year disease assessments under Swiss field conditions (*r* > 0.8). GWAS results identified two significant SNPs associated with anthracnose resistance on gene *Lalb_Chr05_g0216161* encoding a RING zinc-finger E3 ubiquitin ligase which is potentially involved in plant immunity. Population analysis showed a remarkably fast linkage disequilibrium decay, weak population structure and grouping of commercial varieties with landraces, corresponding to the slow domestication history and scarcity of modern breeding efforts in white lupin. Together with 15 highly resistant accessions identified in the resistance assay, our findings show promise for further crop improvement. This study provides the basis for marker-assisted selection, genomic prediction and studies aimed at understanding anthracnose resistance mechanisms in white lupin and contributes to improving breeding programs worldwide.

**Supplementary Information:**

The online version contains supplementary material available at 10.1007/s00122-021-04014-7.

## Introduction

White lupin (*Lupinus albus* L., 2*n* = 50) is a grain legume with a high-protein content and various health benefits that shows great potential to complement soybean and meet the growing demand for plant-based protein (Abraham et al. 2019; Annicchiarico 2008; Lucas et al. 2015). White lupin is believed to originate from the North-Eastern Mediterranean, where wild forms (var. *graecus*) still persist, and has been cultivated for more than 4000 years across the Mediterranean and Eastern Africa (Wolko et al. 2011). Domestication of white lupin has been slow and systematic breeding efforts scarce. In general, lupin species have long been considered valuable assets in crop rotations due to their unique symbiosis with *Bradyrhizobium lupini,* making them highly efficient nitrogen fixers (Fernández-Pascual et al. 2007; Peix et al. 2015). Specifically, white lupin is one of the few crops that form specialized cluster roots, which increase phosphorus availability by carboxylate secretion, significantly increasing soil fertility (Gallardo et al. 2020; Lambers et al. 2013). Since the development of sweet low alkaloid varieties (Kroc et al. 2017), white lupin has become increasingly interesting for the food and feed industry (Lucas et al. 2015).

Cultivation of lupins, however, is severely compromised by the seed- and air-borne fungal pathogen *Colletotrichum lupini*, causing lupin anthracnose (Nirenberg et al. 2002; Talhinhas et al. 2016). Infected seeds (primary infection) and rain-splash dispersal (secondary infection) can cause total yield loss under favorable conditions (Thomas and Sweetingham 2004; White et al. 2008). Typical symptoms are stem twisting and bending and necrotic lesions on stems and pods (Alkemade et al. 2021b). *Colletotrichum lupini* is a member of the *C. acutatum* species complex (clade 1), which contains numerous important plant pathogens (Damm et al. 2012). Contrary to the broad host range seen for most members of this complex, *C. lupini* is host specific to members of the genus *Lupinus* (Baroncelli et al. 2017; Talhinhas et al. 2016). The current lupin anthracnose outbreak started in the 1970s and coincided with a decrease in lupin production worldwide, especially in Europe (FAOSTAT 2021). The pandemic is caused by a globally dispersed and genetically uniform group (II) of highly aggressive strains originating from South America (Alkemade et al. 2021b; Dubrulle et al. 2020a). Little is known about the interaction between *C. lupini* and its host, but a hemibiotrophic lifestyle is considered likely (De Silva et al. 2017; Dubrulle et al. 2020b).

Disease management of anthracnose in white lupin is currently focused on planting pathogen-free seed and chemical control, although the latter strategy is not available for the organic sector and is considered problematic due to adverse environmental effects (Thomas et al. 2008; White et al. 2008). The dispersal of infected symptomless seeds is believed to be the most likely cause of the rapid spread of *C. lupini* strains across the globe. Advanced molecular diagnostics to determine infection levels are developed, but are not yet routinely available (Kamber et al. 2021; Pecchia et al. 2019). Breeding for resistance is therefore likely to be the most sustainable solution. However, no complete resistance has yet been found in white lupin and the trait is considered polygenic (Adhikari et al. 2009; Alkemade et al. 2021a; Jacob et al. 2017). Quantitative trait locus (QTL) mapping of anthracnose resistance using a recombinant inbred line population formed with the highly resistant Ethiopian landrace (P27174) and the susceptible cultivar Kiev Mutant revealed three major resistance QTLs in an Australian experiment (Książkiewicz et al. 2017; Phan et al. 2007; Yang et al. 2010). Unfortunately, accessions selected based on these QTLs did not show increased resistance either under controlled or Swiss field conditions (Alkemade et al. 2021a). The development of a high-throughput phenotyping system for field-relevant anthracnose resistance, together with the availability of a high-quality white lupin reference genome (Hufnagel et al. 2020), allows for more in-depth genomic studies. Genome-wide association studies (GWAS) have proved to be a valuable tool to determine the underlying genetics of quantitative traits in diverse populations and have led to the discovery of single-nucleotide polymorphism (SNP) markers and candidate genes associated with traits of interest for numerous crops (Liu and Yan 2019). As an example, GWAS was recently used with the closely related blue lupin (*L. angustifolius* L.) to identify SNP markers for pod shattering (Mousavi-Derazmahalleh et al. 2018b) and climatic adaptation (Mousavi-Derazmahalleh et al. 2018a).

The aim of this study was to identify SNP markers and candidate genes associated with anthracnose resistance in white lupin. A collection of 200 white lupin cultivars, breeding lines and landraces, originating from across the Mediterranean and important cultivation regions, was genotyped-by-sequencing (GBS) and phenotyped for anthracnose resistance under controlled conditions. These accessions were shown to be variable for key agronomic traits (Annicchiarico et al. 2010) such as drought tolerance (Annicchiarico et al. 2018) and grain yield (Annicchiarico et al. 2019). Understanding the genetic architecture of anthracnose resistance in white lupin will provide crucial information to support further crop improvement.

## Material and methods

### Germplasm collection

White lupin (*Lupinus albus* L.) accessions were collected across the Mediterranean region, Atlantic islands, Eastern Africa, Europe, Chile and Australia from seed genebanks and local partners. The accessions are described in Electronic Supplemental Material 1 (ESM_1). In total, the 200 accessions include commercial cultivars, breeding lines and traditional land races. The collection includes a large number of landraces from CREA’s white lupin world collection (Annicchiarico et al. 2010), widely studied accessions such as Amiga, Feodora, Kiev Mutant and P27174 (Adhikari et al. 2009; Hufnagel et al. 2021), and recently discovered anthracnose-resistant lines (Alkemade et al. 2021a).

### Disease phenotyping

The white lupin collection was phenotyped for anthracnose resistance under controlled conditions (25 ± 2 °C, 16 h light and 70% relative humidity) using the high-throughput protocol described by Alkemade et al. (2021a). Stem wound inoculations were performed with the highly virulent *Colletotrichum lupini* strain JA01 (genetic group II; Alkemade et al. (2021b)). Disease was assessed at 3, 7 and 10 days post-inoculation (dpi) with a 1 to 9 disease score index (DSI), with 1 being healthy and 9 completely diseased (Alkemade et al. 2021a). At 10 dpi, lesion size, stem length and shoot fresh weight were determined. The overall disease score (based on the three DSI assessments) is expressed as the standardized area under the disease progress curve (sAUDPC, Jeger and Viljanen-Rollinson (2001)), the lesion size as relative to overall stem length (LS_rel_), and the shoot fresh weight is expressed relative to a control (SFW_rel_). All of the experiments were performed in a randomized complete block design with a minimum of 8 replicates per accession.

### Field trials

Phenotypic data obtained under controlled conditions of twelve selected accessions, ranging from susceptible to resistance, were compared to phenotypic data of these twelve accessions acquired over three-year field trials in Switzerland (ESM_1). Field trials were performed within six row plots according to Alkemade et al. (2021a) at three distinct sites: Mellikon (47°34′05.3"N 8°21′19.3″E) in 2018 and 2019, Full-Reuenthal (47°36′02.8″N 8°11′35.2″E) in 2020 and Feldbach (47°14′20.0″N, 8°47′18.8″E) in 2018, 2019 and 2020. Trials performed in 2018 and 2019 are described in Alkemade et al. (2021a). In 2020, in Full-Reuenthal plot sizes were 1.32 × 3.5 m and in Feldbach plot sizes were 1.5 × 2.7 m with a seed density of 65 seed/m^2^. Total field size was 304 m^2^ in Full-Reuenthal and 259 m^2^ in Feldbach. Trials were performed in a randomized complete block design consisting of 4 replicates. The field trials relied on natural infection and were scored 80, 100 and 135 days after sowing. The DSI ranged from 1 (healthy) to 9 (dead), as described in Alkemade et al. (2021a). The sAUDPC and yield (dt/ha) were determined.

### Phenotypic data analysis

Statistical analyses of the phenotypic data were performed within R 4.0.3 (R Core Team 2020) using the packages *lme4* (Bates et al. 2015)*, lmerTest* (Kuznetsova et al. 2017) and *emmeans* (Lenth et al. 2019)*,* following a mixed model. The factors of interest (i.e., accession) were included as fixed effects, while environment, environment x accession and replicated block nested in environment were fitted as random factors, after confirming the assumptions of normality of residuals and homogeneity of variance. To achieve a normal distribution, data were transformed with a square root (yield), log10 (SFW_rel_), square (sAUDPC_CC_), or logit (LS_rel_) transformation. The mean separation between accessions and the overall mean of all accessions combined was analyzed using Dunnett’s test (*P* ≤ 0.05). The data are presented as non-transformed estimated least-squares means obtained using the aforementioned mixed model. Estimated means of controlled and field conditions were correlated using the Pearson correlation coefficient. Broad sense heritability (H^2^) was estimated as: genotypic variance/phenotypic variance (Toker 2004).

### Genotyping and SNP calling

Genomic DNA was isolated from leaf tissue of three week old plants using DNeasy Plant Mini Kit (Qiagen, Hilden, D) and quantified with a Quant-iT™ PicoGreen™ dsDNA Assay Kit (Thermo Fisher Scientific, Waltham, MA, USA). Samples were genotyped in four different batches referred to as TX2016-1 (88 samples), TX2016-2 (32 samples), EL2018 (40 samples) and EL2020 (40 samples), using slightly different procedures as follows:

Genotyping-by-sequencing (GBS) libraries for TX2016-1 and TX2016-2 were prepared with a modified Elshire et al. (2011) protocol. DNA samples (100 ng) were digested with restriction enzyme *ApeKI* (New England Biolabs, Ipswich, MA, USA) and ligated to unique barcodes and common adapters. Equal volumes of ligated products were pooled and purified with NucleoSpin Gel and PCR Clean-up (Macherey–Nagel, Düren, D). Template DNA (50 ng) was mixed with two primers (ESM_2) and KAPA Library Amplification Readymix (Roche, Basel, CH). Amplification steps were as follows: 5 min at 72 °C, 30 s at 98 °C and 10 cycles with 10 s at 98 °C, 30 s at 65 °C and 30 s at 72 °C. Sequencing was performed at the University of Texas (USA) on four Illumina HiSeq 2000 (Illumina Inc., San Diego, CA, USA) lanes, at 100 bp single end. DNA samples for libraries EL2018 and EL2020 were sent to The Elshire Group Ltd. (Palmerston North, New Zealand) for library preparation and sequencing. Library preparation was performed according to Elshire et al. (2011) as described above with the following changes: libraries were amplified with 14 PCR cycles and prepared using a KAPA HyperPrep Kit (Roche, Basel, CH) following the manufacturer’s instructions. Sequencing was performed on a single Illumina HiSeq X lane, at 2X150 bp paired end.

GBS raw reads were demultiplexed using axe demultiplexer (Murray and Borevitz 2018). Trimming for restriction enzyme remnants, alignment on reference genome and SNP calling were performed using the dDocent pipeline (Puritz et al. 2014). For alignment we used the *L. albus* genome version 1.0 (Hufnagel et al. 2020) which was downloaded from https://www.whitelupin.fr/. The final genotype matrix, in the form of a vcf file, was further filtered for quality using the vcftools software (Danecek et al. 2011) with parameters − minQ 30 − max-non-ref-af 1 –non-ref-af 0.001. The resulting data set of 246,279 SNPS was filtered for monomorphic markers, minor allele frequency (MAF) < 5%, missing SNP marker rate > 10%, and a missing rate per individual > 20% (Pavan et al. 2020). Genotypes that deviated with 3 SD from the mean heterozygosity rate were removed (Marees et al. 2018). Missing data were imputed through Beagle (Browning and Browning 2016) within *statgenGWAS* (van Rossum et al. 2020) in *R*, resulting in 4611 high-quality SNPs for 181 accessions (ESM_3).

### Linkage disequilibrium and population structure

Linkage disequilibrium (LD) of SNP markers was calculated as the pairwise squared correlation coefficient (*r*^2^) between markers using *LD.decay* in *R* (Laido et al. 2014). Significant (*P* ≤ 0.05) pair-wise LD estimates were used to calculate average LD decay within a sliding window of 5 kb. LD decay was visualized by plotting *r*^2^ estimates against genetic distance (kb). A pairwise distance matrix derived from Euclidean distance of the full SNP dataset was calculated in R to construct a Ward Hierarchical clustering tree (Murtagh and Legendre 2014) with 1000 bootstraps using *pvclust* in R (Suzuki and Shimodaira 2006). The tree was generated with *ape* (Paradis and Schliep 2019) in R and modified in iTOL v 6.1 (Letunic and Bork 2007). Principal component analysis (PCA) was performed using the *prcomp* function in R based on 1,292 SNPs filtered for a MAF > 20 and physical distance > 2.5 kb. An Astle kinship matrix (Astle and Balding 2009) was generated using *statgenGWAS* in R using the pruned SNP dataset.

### Genome-wide association mapping

A genome-wide association study (GWAS) was performed on 181 accessions and 4,611 SNPs using estimated least-square means for the traits disease score (sAUDPC), LS_rel_ and SFW_rel_. The association between SNPs and phenotypes was determined by performing a single-trait GWAS following a single-locus mixed linear model (MLM) and multi-locus Bayesian information and linkage disequilibrium iteratively nested keyway (BLINK) model (Huang et al. 2019). MLM was performed within *statgenGWAS* in R following the method described in Kang et al. (2010). The first ten principal components (PCs) were included as covariates to control for population structure, and the Astle kinship matrix was included to account for cryptic relatedness (Astle and Balding 2009; Rincent et al. 2014). An efficient mixed model association (EMMA) algorithm was used to estimate the variance components (Kang et al. 2008). General least squares (GLS) were used to estimate effect size and P-value for each SNP. BLINK was performed within GAPIT 3.0 (Wang and Zhang 2021) using the first ten PCs. A Bonferroni corrected LOD threshold ( − log10(0.05/number of SNPs)) was used to identify significant SNPs, and a fixed threshold of − log10(5.00E-05)) was used to identify low peak SNPs otherwise missed by the highly conservative Bonferroni threshold (Storey and Tibshirani 2003). SNPs within 2.5 kb and ≥ 0.5 *r*^2^ were considered linked. Manhattan and quantile–quantile (Q–Q) plots were generated within *statgenGWAS*.

### Candidate gene selection

Candidate genes were considered when containing a significant SNP or a SNP in LD (*r*^2^ > 0.5) with a significant SNP and when within 10 kb of significant SNPs. Candidate genes were located using the white lupin reference genome (v 1.0) browser (Hufnagel et al. 2020). Protein sequences were acquired and blasted (BLASTp) to find homologs in closely related species, i.e., blue lupin (*L. angustifolius*), peanut (*Arachis hypogea*), common bean (*Phaseolis vulgaris*), soybean (*Glycine max*) and model species *Medicago truncatula* (LPWG 2017). The potential function of each candidate gene was derived from annotations, literature and in silico analysis.

## Results

### Strong differentiation and heritability of anthracnose-related traits

**Fig. 1:**
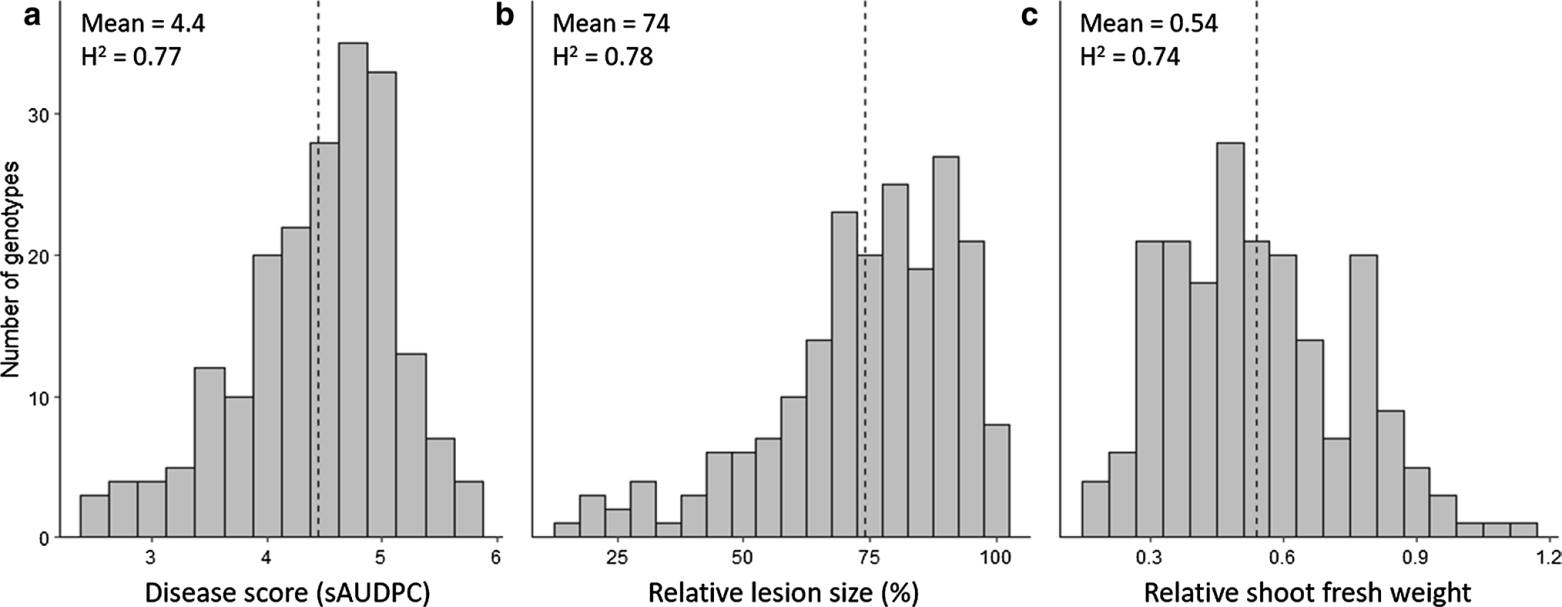
Phenotypic variation in three anthracnose resistance traits among 200 white lupin accessions. **a** Disease score (standardized area under the disease progress curve (sAUDPC)). **b** Relative lesion size (%). **c** Relative shoot fresh weight. Dashed line indicates overall mean

Disease phenotyping of 200 white lupin accessions under controlled conditions revealed a range of resistant and susceptible accessions (Fig. 1). Strong differences between accessions (*P* < 0.0001) for all three anthracnose related traits, sAUDPC, LS_rel_ and SFW_rel_, were observed with heritabilities of 0.77, 0.78 and 0.74, respectively. Significant (*P* ≤ 0.05) Pearson correlations were found between the three traits (*r* > (−)0.86; ESM_4). For twelve selected accessions, strong correlations were observed between these three traits and the overall disease assessment means of three-year field trials in Switzerland (r > (−)0.8; ESM_5). We did not observe complete resistance against anthracnose, which we interpret as evidence that resistance in white lupin is quantitative. For sAUDPC, LS_rel_ and relative SFW_rel_, 8, 13 and 2 accessions, respectively, were more resistant than the respective overall mean (*P* ≤ 0.05, ESM_1). Six of these accessions originated from Ethiopia, two from Chile and one being the newly available commercial variety Frieda. In contrast, seven accessions were more susceptible than the overall mean. Remarkably, the Ethiopian landrace P27175 was resistant in one seed batch (FiBL 39) but susceptible in another (FiBL 19) and showed distinct heterozygosity rates of 0.77% and 32.2%, respectively (ESM_1). Resistant FiBL025 (R-6020) and susceptible LAP0119b (Aster) seeds showed a black-speckled morphology typical for wild *graecus* types.

### Weak population structure and fast LD decay

**Fig. 2:**
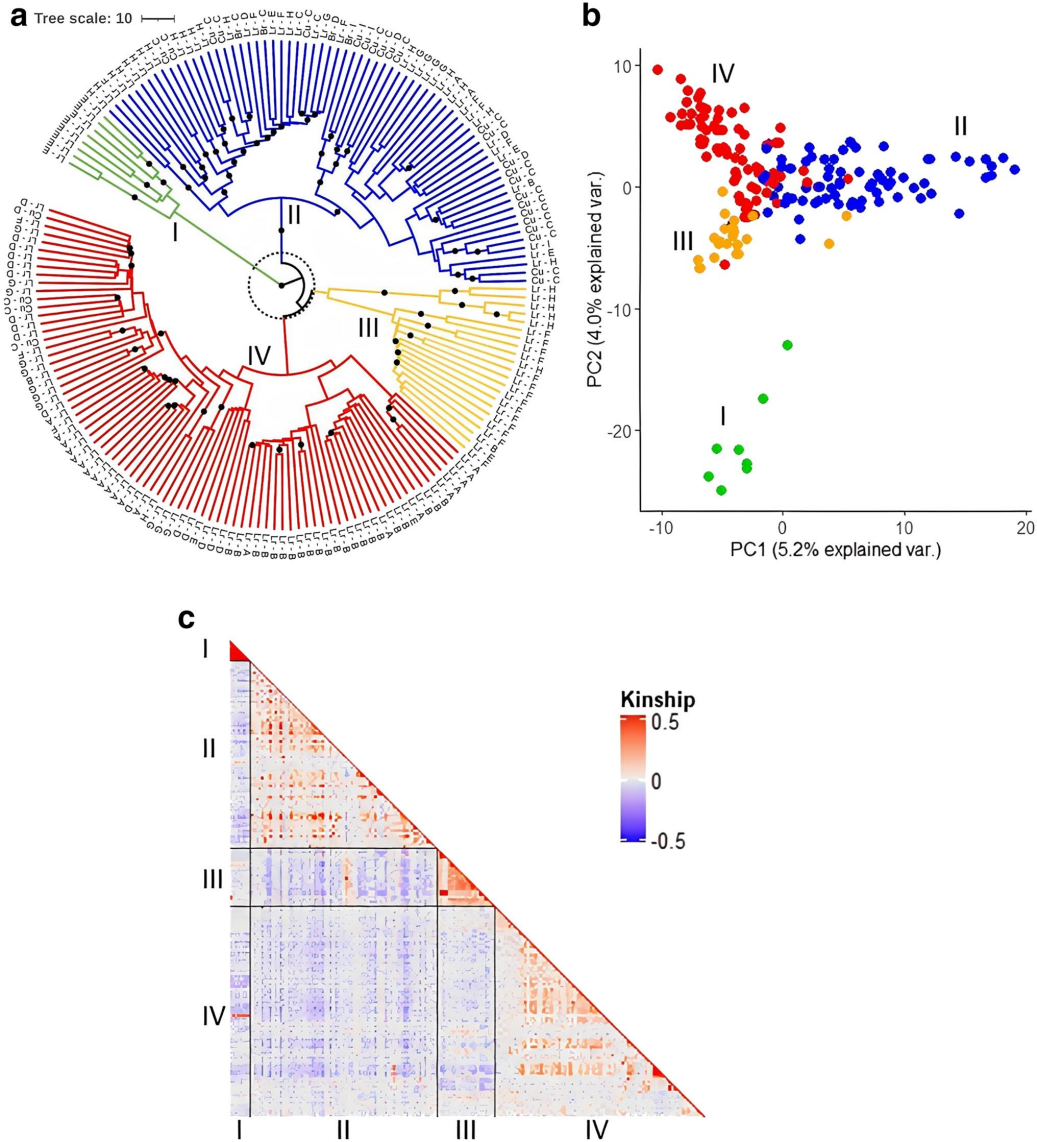
Genetic diversity and population structure of 181 white lupin accessions. **a** Ward cluster analysis (1,000 bootstraps). Colors represent subgroups (I-IV). Black dots represent bootstrap support values (> 90), and dotted circle indicates branch length of 10. Letters indicate accession type (inner) and region of origin (outer) with Lr = landrace, Cu = cultivar, Br = breeding line, A = Atlantic Isles, B = Iberian Peninsula, C = West and Eastern Europe, D = North-East Mediterranean, E = South-East Mediterranean, F = Egypt, G = South-West Mediterranean, H = East Africa and I = Chile. **b** Principal component analysis (PCA). Each dot represents an accession and colors represent subgroups (I-IV). **c** Heatmap of the Astle kinship value among accessions

Genotyping by sequencing yielded 4611 high-quality SNPs for 181 accessions after filtering for monomorphic markers, minor allele frequency (MAF) < 5%, missing SNP marker rate > 10%, missing rate per accession > 20% and heterozygosity > 40% (mean Ho = 15%; ESM_1). LD decayed to half its maximum value at 2.9 kb (*r*^2^ = 0.45), and SNPs were in linkage (*r*^2^ > 0.5) over an average distance of 2.5 kb (ESM_6). Cluster analysis on the full SNP dataset distinguished 4 subgroups (I–IV) based on bootstrap support values (BS) > 90 and a branch length threshold of 10 (Fig. 2a). These subgroups could also be observed through PCA and Astle kinship analysis after pruning the SNP dataset (MAF > 20% and physical distance > 2.5 kb; Fig. 2b and c), which revealed overlap between groups II, III and IV, while group I was more clearly separated. Group I exclusively contained landraces originating from the South-East Mediterranean (Fig. 2a and ESM_1). Group II, which includes accessions from across the entire study area, encompasses most of the commercial cultivars and breeding lines used in this study (88%). A large proportion (27%) of group II includes landraces from North Africa, with half originating from Ethiopia. Group III consists mostly of Egyptian (64%) and Ethiopian (23%) landraces, with the Ethiopian landraces strongly clustering together (BS = 100). Group IV contains accessions from across the entire study area, including lines from the Iberian Peninsula (30%) and the Atlantic Isles (26%). Kinship between accessions showed relatively close relatedness among sampled accessions (Fig. 2c).

### Three significant SNPs associated with anthracnose resistance

**Fig. 3:**
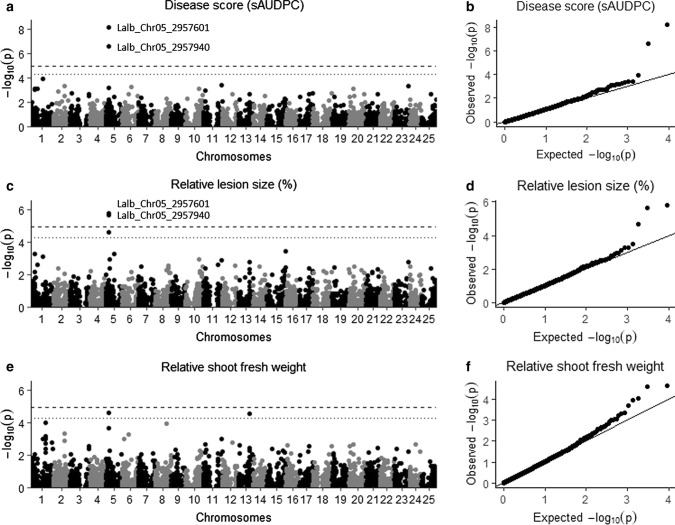
Manhattan and corresponding Q–Q plots by MLM showing SNP association with anthracnose resistance. **a, b** Disease score (standardized area under the disease progress curve (sAUDPC)). **c, d** Relative lesion size (%). **e, f** Relative shoot fresh weight. Upper dashed line indicates Bonferroni corrected LOD threshold of 4.96 (*P* = 1.08E-05), and lower dotted line indicates fixed LOD threshold of 4.3 (*P* = 5.00E-05)

**Fig. 4:**
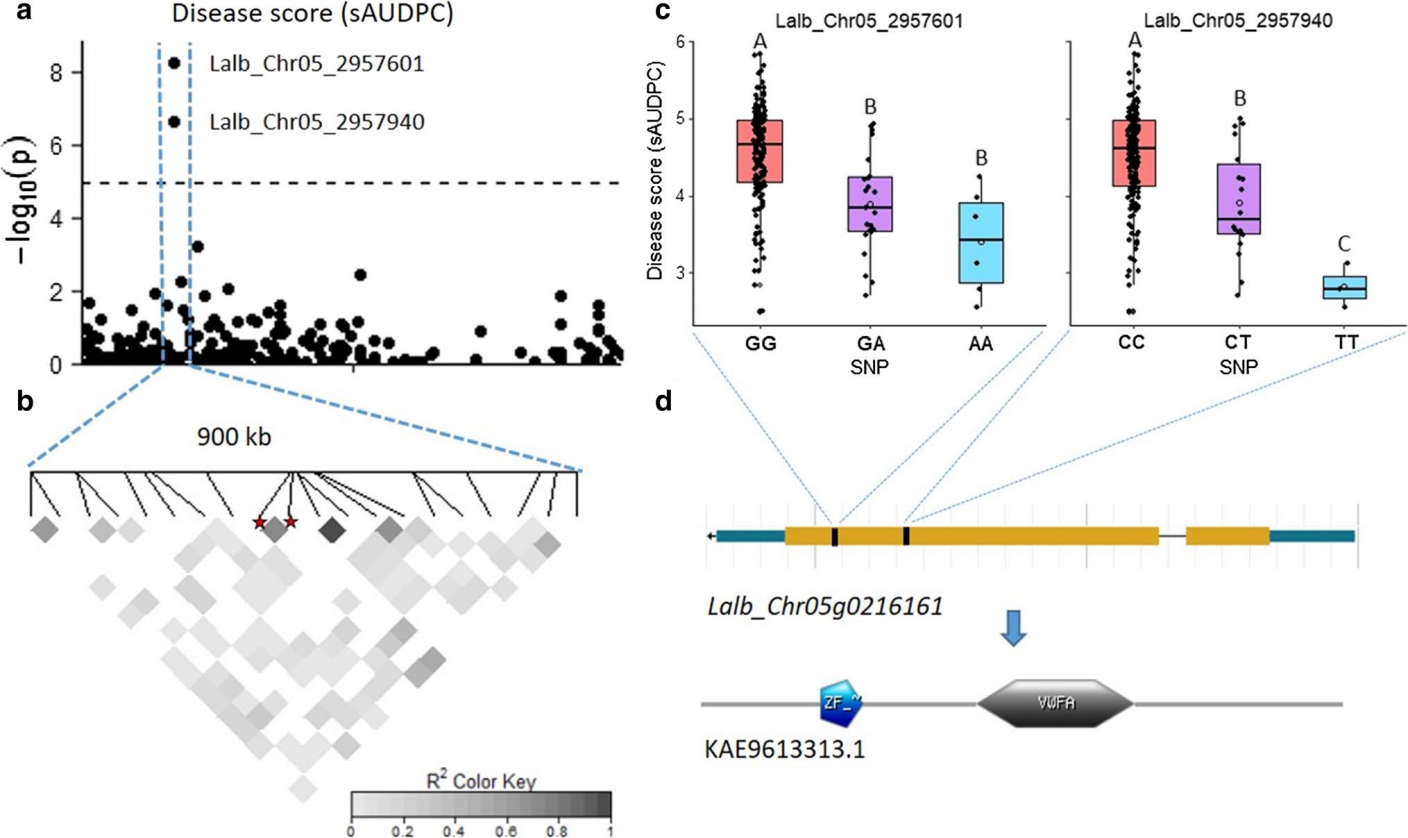
Characterization of SNP Lalb_Chr05_2957601 & -940. **a** Manhattan plot of chromosome 5, dashed line indicates Bonferroni corrected LOD threshold of 4.96 (*P* = 1.08E-05). **b** Linkage disequilibrium (LD) heatmap of 20 SNPs within 500 kb of significant SNPs (stars). **c** Boxplots showing allele effect on disease score (standardized area under the disease progress curve (sAUDPC)). Capital letters within plot indicate significant difference (Tukey-HSD, *P* ≤ 0.05). **d** Candidate gene *Lalb_Chr05g0216161*, showing protein coding region (orange) and spliced non-coding RNA (blue; www.whitelupin.fr) and corresponding protein KAE9613313.1 with Von Willebrand factor type A (VWFA) and RING zinc-finger domain (ZF; De Castro et al. (2006))

To map genetic variants associated with anthracnose resistance, we performed a genome-wide association study (GWAS) following a MLM and BLINK model. We included the first 10 principal components (PCs) and the Astle kinship matrix to correct for population structure. The first 10 PCs explained 24% of the genetic variance. The resulting Q-Q plots revealed that the model was well calibrated, as we observed a good approximation between the expected and observed P-values (Fig. 3 and ESM_7). Following the MLM and applying a Bonferroni LOD-threshold of 4.96 (*P* = 1.08E-05), we identified two highly significant SNPs, Lalb_Chr05_2957601 and Lalb_Chr05_2957940, for both sAUDPC (*P* = 6.11E-09 and 2.41E-07, respectively) and LS_rel_ (*P* = 1.55E-06 and 2.13E-06, respectively; Fig. 3 and Table 1). Analysis with the BLINK model identified a significant association between Lalb_Chr05_2957601 and sAUDPC (*P* = 6.58E-12) and SFW_rel_ (*P* = 2.72E-07), and between Lalb_Chr05_2957940 and LS_rel_ (*P* = 2.72E-07; ESM_7 and Table 1). The two SNPs explained 12 to 16% of the observed variation (*R*^2^_LR_; Table 1), were not strongly linked with other SNPs and were found in exons of the same gene (*Lalb_Chr05g0216161*; Fig. 4). The minor allele frequencies (MAF) of these SNPs were 10 and 7%, respectively, and the non-reference alleles were significantly associated with increased anthracnose resistance when either heterozygous or homozygous (Fig. 4c). The non-reference alleles were only found homozygous for both SNPs in the Chilean accessions Fibl016 (Blu-25) and LAP0155a & b (Rumbo Baer), which were found to be highly resistant (sAUDPC = 2.55, 2.78 and 3.11, respectively).

**Table 1:** Significant SNPs and associated candidate genes

Trait	SNP^a^	Allele	*P* value MLM	*P* value BLINK	R^2^_LR_	MAF	Candidate gene	Annotation
Disease Score (sAUDPC)	Lalb_Chr05_2957601	G/A	6.11E-09	6.58E-12	0.16	0.10	*Lalb_Chr05 g0216161*	E3 ubiquitin-protein ligase
	Lalb_Chr05_2957940	C/T	2.41E-07	0.016^b^	0.13	0.07		
Relative lesion size (%)	Lalb_Chr05_2957601	G/A	1.55E-06	0.042^b^	0.12	0.10		
	Lalb_Chr05_2957940	C/T	2.13E-06	3.98E-10	0.12	0.07		
Relative shoot fresh weight	Lalb_Chr05_2957601	G/A	2.29E-05^b^	2.72E-07	0.09	0.10		
	Lalb_Chr05_2957940	C/T	1.81E-04^b^	0.11^b^	0.07	0.07		
Relative lesion size (%)	Lalb_Chr05_3706534	A/G	2.21E-05^b^	1.91E-07	0.10	0.16	*Lalb_Chr05 g0217341*	Paired amphipathic helix protein Sin3-like 4

*SNP* single-nucleotide polymorphism, *MAF* minor allele frequency, *R*^2^_LR_ likelihood-ratio-based *R*^2^, *sAUDPC* standardized area under the disease progress curve.

^a^*Lalb* *Lupinus albus*, *Chr* chromosome, number = position on chromosome. ^b^Not significant

Analysis with the BLINK model identified an additional SNP on chromosome 5, Lalb_Chr05_3706534 (*P* = 1.91E-07), associated with LS_rel_ (ESM_7 and Table 1). This SNP explained 10% of the observed variation and showed a MAF of 16%. The non-reference allele of this SNP was implicated in decreased anthracnose resistance (ESM_8). SNPs Lalb_Chr05_3688076 and Lalb_Chr05_3784474 were considered linked with Lalb_Chr05_3706534 (ESM_9). Other promising SNPs, above the fixed LOD threshold of 4.3 (*P* = 5.00E–5)), were found on chromosome 1 (Lalb_Chr01_3872625) for LS_rel_, on chromosome 13 (Lalb_Chr13_12108967) for SFW_rel_ and on chromosome 6 (Lalb_Chr06_9655085) for sAUDPC (ESM_9).

### Candidate genes involved in resistance pathways

Candidate genes were considered when containing a significant SNP, a SNP linked (*r*^2^ > 0.5) to a significant SNP or when located within 10 kb of a significant SNP. The significant SNPs Lalb_Chr05_2957601 and Lalb_Chr05_2957940 are both located within an exon of the same gene: *Lalb_Chr05g0216161* (Table 1, Fig. 4d). This gene is annotated as a putative chromatin regulator and encodes a protein containing a Von Willebrand factor type A (VWFA) as well as a RING zinc-finger domain (ZF; Fig. 4d). Homologs in closely related legume species encode for RING zinc-finger E3 ubiquitin ligases, which are widely associated with plant immunity (Marino et al. 2012). Lalb_Chr05_3706534 is located within an intron of gene *Lalb_Chr05g0217341* which encodes a putative transcription regulator, and homologs in related species encode a paired amphipathic helix protein Sin3. Linked to this SNP is Lalb_Chr05_3784474, which is located within an exon of the gene *Lalb_Chr05g0217471* which encodes for a non-specific serine/threonine protein kinase (ESM_9). Homologs in closely related species encode leucine-rich repeat (LRR) receptor-like serine/threonine-protein kinases, which are often implicated in plant defense against fungal pathogens (Afzal et al. 2008; Tang et al. 2017).

## Discussion

Disease phenotypes of twelve selected accessions obtained under controlled conditions, strongly correlated (*r* > 0.8) to overall three-year field plot disease assessments in Switzerland, confirming field-relevance of high-throughput phenotyping under controlled conditions (Alkemade et al. 2021a). No complete resistance was observed, but based on sAUDPC, LS_rel_ and SFW_rel_, a total of 15 different accessions showed to be significantly more resistant to anthracnose compared to the overall mean. High resistance was found for Chilean cultivar Rumbo Baer and the breeding line Blu-25, both of which appear to derive from a resistant landrace from the Azores (von Baer et al. 2009). Five resistant accessions originated from Ethiopia, which was previously shown to be a good source for white lupin anthracnose resistance (Adhikari et al. 2009; Cowling et al. 1999). However, Ethiopian landrace P27174 (Fibl020 & 38), used as a resistant parent for an anthracnose resistance QTL study (Książkiewicz et al. 2017; Yang et al. 2010), was not shown to be resistant in this study and in Alkemade et al. (2021a), suggesting the occurrence of cross-pollination or admixture. Resistance and heterozygosity rate in Ethiopian landrace P27175 differed among seed batches, suggesting differences in seed quality, outcrossing or that P27175 represents a mixture of genotypes. These observed irregularities for P27174 and P27175 should be further investigated.

White lupin has long been cultivated across the Mediterranean and North-Eastern Africa, with its primary center of origin believed to be in the Balkans up to Western Turkey where wild *graecus* types are still found (Wolko et al. 2011). This study, which contains accessions collected from across the traditional cultivation regions of white lupin, revealed an exceptionally fast LD decay (2.9 kb). This fast LD decay is consistent with an earlier study by Hufnagel et al. (2021) and the fact that white lupin has a modest rate of outcrossing (Green et al. 1980). Studies on other grain legume species, including pea (> 50 kb; Gali et al. (2019)), soybean (> 240 kb; Wen et al. (2018)), and common bean (> 1 Mb; Diniz et al. (2019)), as well as the closely related blue lupin (> 77 kb; Mousavi-Derazmahalleh et al. (2018b)), have all reported slower rates of LD decay. The analyzed population showed an average heterozygosity rate of 15%, which is similar to observed outcrossing rates of 10% (Green et al. 1980). The heterozygosity within the studied population could have reduced the power of the GWAS to detect major loci (Alqudah et al. 2020). Increasing sample size, marker coverage and creating an inbred population could improve the analysis.

In addition, we detected a weak population structure, finding four subgroups (I–IV). Principal component analysis showed overlap between Groups II, III and IV, while only Group I, exclusively containing landraces from the South-Eastern Mediterranean, formed a clearly distinct group. Group III consisted primarily of Egyptian and Ethiopian landraces, with Ethiopian accessions strongly grouping together. Landraces from Ethiopia were previously reported to form a distinct group within white lupin (Raman et al. 2014) and were shown to be most closely related to wild *graecus* types (Hufnagel et al. 2020, 2021), which suggests these landraces derived in isolation and are still little domesticated. In contrast with these results, 10 of the 16 Ethiopian landraces collected in this study were classified in Group II, containing commercial cultivars and landraces from all across the collection area. Taken together, we interpret the fast LD decay, weak population structure and the grouping of commercial varieties with landraces to indicate that there have been few recent breeding events in white lupin, which implies that there is great potential for further crop improvement of this re-emerging protein crop.

GWAS analysis by MLM and BLINK identified three significant SNPs, Lalb_Chr05_2957601, 2957940 and 3706534, on chromosome 5 associated with anthracnose resistance. Additionally, three SNPs were identified above the fixed LOD threshold of 4.3 on chromosomes 1, 6 and 13. Compared to MLM, BLINK improved statistical power and removed redundant genetically linked SNPs (Huang et al. 2019). It should be considered, however, that the genome-wide marker coverage in this study might not have been adequate to replace the polygenic effect of the kinship matrix and population structure by covariate markers as done in multi-locus methods such as BLINK (Tibbs Cortes et al. 2021). The identified SNPs do not correspond to previously reported QTLs associated with anthracnose or phomopsis (*Diaporthe toxica*) resistance in white lupin (Cowley et al. 2014; Książkiewicz et al. 2017). Corresponding candidate genes also do not reflect anthracnose resistance genes identified in blue lupin, including *Lanr1, Anman* and *LanrBo* (Fischer et al. 2015; Yang et al. 2004, 2008).

SNPs Lalb_Chr05_2957601 and 2957940 are both located in the same coding region of *Lalb_Chr05g0216161* which encodes for a protein with a RING zinc-finger and VWFA domain. The MAF for these SNPs was low, and non-references alleles were only homozygous in Chilean, Ethiopian and Moroccan accessions, but were also present in wild *graecus* types (LD37, GR38, and Batsi; Hufnagel et al. (2021)). Homologs in closely related legume species encode RING zinc-finger E3 ubiquitin-protein ligases. E3 ubiquitin-ligases have frequently been shown to be involved in different steps of plant immunity (Duplan and Rivas 2014; Marino et al. 2012; Zhou and Zeng 2017). In pepper (*Capsicum annuum*), the RING finger protein gene, *CaRFP1*, containing a VWFA domain, was shown to act as E3 ubiquitin ligase and was highly upregulated during *C. coccodes* infection (Hong et al. 2007). Other RING type E3 ubiquitin ligases were shown to influence resistance against *Magnaporthe oryzae* in rice (Park et al. 2016), *Xanthonomas* infection in *C. annuum* (Lee et al. 2011) and *Ralstonia solanacearum* in tobacco (Ghannam et al. 2016). Besides biotic stress, RING E3 ubiquitin ligases have shown to improve resistance against abiotic stresses (Cho et al. 2017; Lee and Kim 2011), such as drought (Cheng et al. 2012) and salt stress (Kim and Kim 2013), and were shown to be involved in various plant developmental processes (Shu and Yang 2017), such as root development (Sakai et al. 2012). In conclusion, the identified gene, *Lalb_Chr05g0216161*, might play an important role in anthracnose resistance in white lupin and should be further investigated.

SNP Lalb_Chr05_3706534 is located in the non-coding region of *Lalb_Chr05g0217341.* Homologs of *Lalb_Chr05g0217341* in closely related legume species encode for paired amphipathic helix protein Sin3-like 4 proteins, which are known to be involved in powdery mildew (*Podosphaera fusca*) resistance in cucumber (Liu et al. 2021). SNP Lalb_Chr05_3784474 is considered linked to Lalb_Chr05_3706534 and is located within the gene *Lalb_Chr05g0217471* which encodes a LRR containing receptor-like protein with a serine/threonine kinase. LRR receptor kinases are well known as resistance genes and for their role in plant immunity (Afzal et al. 2008; Ellis et al. 2000; Tang et al. 2017), and serine/threonine kinases were shown to be involved in signaling during pathogen recognition and subsequent activation of plant defense mechanisms (Afzal et al. 2008; Goff and Ramonell 2007). LRR receptor-like serine/threonine-protein kinases were shown to confer resistance against apple scab (*Venturia inaequalis*) in apple (Padmarasu et al. 2018) and against rice blast (*Magnaporthe grisea*) in rice (Song et al. 2008). The fact that the three significant SNPs, representing two loci (*Lalb_Chr05g0216161* and *Lalb_Chr05g0217341*), could only explain 16 to 34% of disease phenotypic variance, the identification of few low peak SNPs and the quantitative nature of the trait suggests that anthracnose resistance is under polygenic control as indicated by Książkiewicz et al. (2017), but the identification of one major loci (*Lalb_Chr05g0216161*) could also imply oligogenic control.

This study showed that GWAS, thanks to the weak population structure, fast LD decay and the availability of a high-quality reference genome, is a powerful tool to identify resistance loci in white lupin and provides the basis for further gene mapping. Further characterization of the E3 ubiquitin ligase encoding *Lalb_Chr05g0216161* gene, e.g., via gene expression studies, could shed first light on white lupin resistance mechanisms against anthracnose disease. The obtained dataset also provides a basis for marker-assisted selection and the development of genomic prediction models for anthracnose resistance. Overall, this study contributes to understanding the genetic make-up of anthracnose resistance in white lupin and supports future crop improvement.

## Supplementary Information

Below is the link to the electronic supplementary material.

Supplementary file1 (PDF 998 kb)

Supplementary file2 (XLSX 38 kb)

Supplementary file3 (XLSX 2751 kb)

## Data Availability

The datasets generated during the current study are available at: 10.5281/zenodo.5142130.
