# Supplementary material for: Genome-wide association study reveals white lupin candidate gene involved in anthracnose resistance
Source: Theor Appl Genet. 2022 Jan 5;135(3):1011–24. doi: 10.1007/s00122-021-04014-7 (PMC8942938; doi:10.1007/s00122-021-04014-7)
Supplement: Supplementary file 1 — Supplementary file1 (PDF 998 kb) [file 122_2021_4014_MOESM1_ESM.pdf]

Article Title

Genome-wide association study reveals white lupin candidate gene involved in anthracnose resistance

Journal

Theoretical Applied Genetics

Authors

Joris A. Alkemade[1], Nelson Nazzicari[2], Monika M. Messmer[1]*, Paolo Annicchiarico[2], Barbara Ferrari[2], Ralf T. Voegele[3], Maria R. Finckh[4], Christine Arncken[1], Pierre Hohmann[1]

1 Department of Crop Sciences, Research Institute of Organic Agriculture (FiBL), Frick, Switzerland
2 CREA, Research Centre for Animal Production and Aquaculture, Lodi, Italy
3 Institute of Phytomedicine, University of Hohenheim, Stuttgart, Germany
4 Department of Ecological Plant Protection, University of Kassel, Witzenhausen, Germany

* Corresponding author:

Email: monika.messmer@fibl.org

ESM_2: Primers used for library preparation.

Primers	Sequence
PCRprimer1	AAT GAT ACG GCG ACC ACC GAG ATC TAC ACT CTT TCC CTA CAC GAC GCT CTT CCG ATC T
PCRprimer2	CAA GCA GAA GAC GGC ATA CGA GAT CGG TCT CGG CAT TCC TGC TGA ACC GCT CTT CCG ATC T

ESM_4: Correlation between disease assessments under controlled and three-year Swiss field conditions [a].

	sAUDPC Field	Yield (dt/ha) Field	sAUDPC CC	LS_{rel} CC	SFW_{rel} CC
**sAUDPC Field**	1.00				
**Yield (dt/ha) Field**	-0.79*	1.00			
**sAUDPC CC**	0.86*	-0.56•	1.00		
**LS_{rel} CC**	0.87*	-0.61*	0.96*	1.00	
**SFW_{rel} CC**	-0.80*	0.53•	-0.92*	-0.86*	1.00

[a] sAUDPC = standardized area under the disease progress curve, LS_{rel} = relative lesion size (%), SFW_{rel} = relative shoot fresh weight, CC = controlled conditions, correlations are expressed as Pearson correlation coefficients. * $P \leq 0.05$, • $P \leq 0.1$.

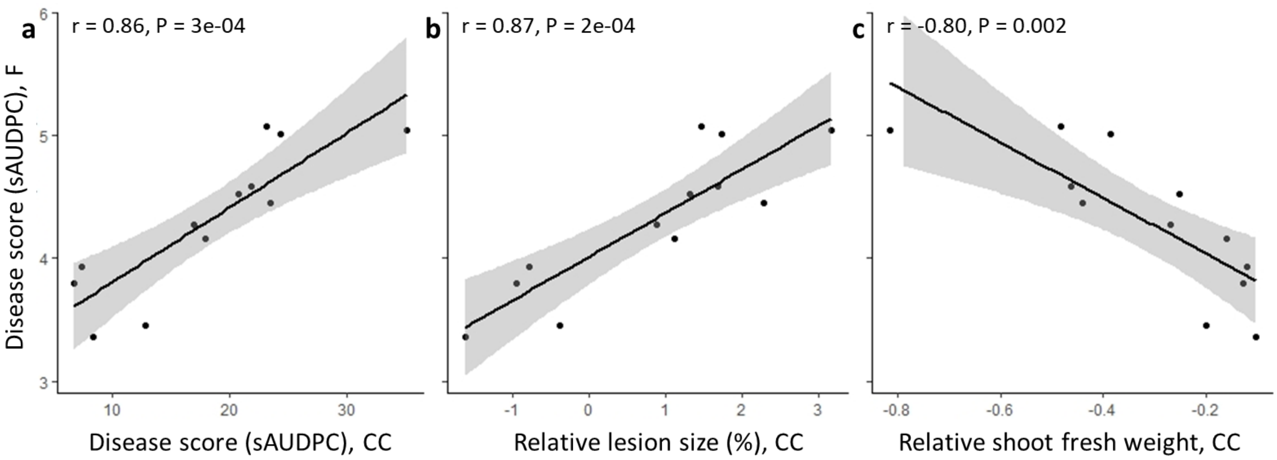

ESM_5: Correlations between anthracnose-related traits assessed under controlled conditions and three-year averaged disease scores assessed in the field. CC = controlled conditions, F = field, sAUDPC = standardized area under the disease progress curve, r = Pearson correlation coefficient.

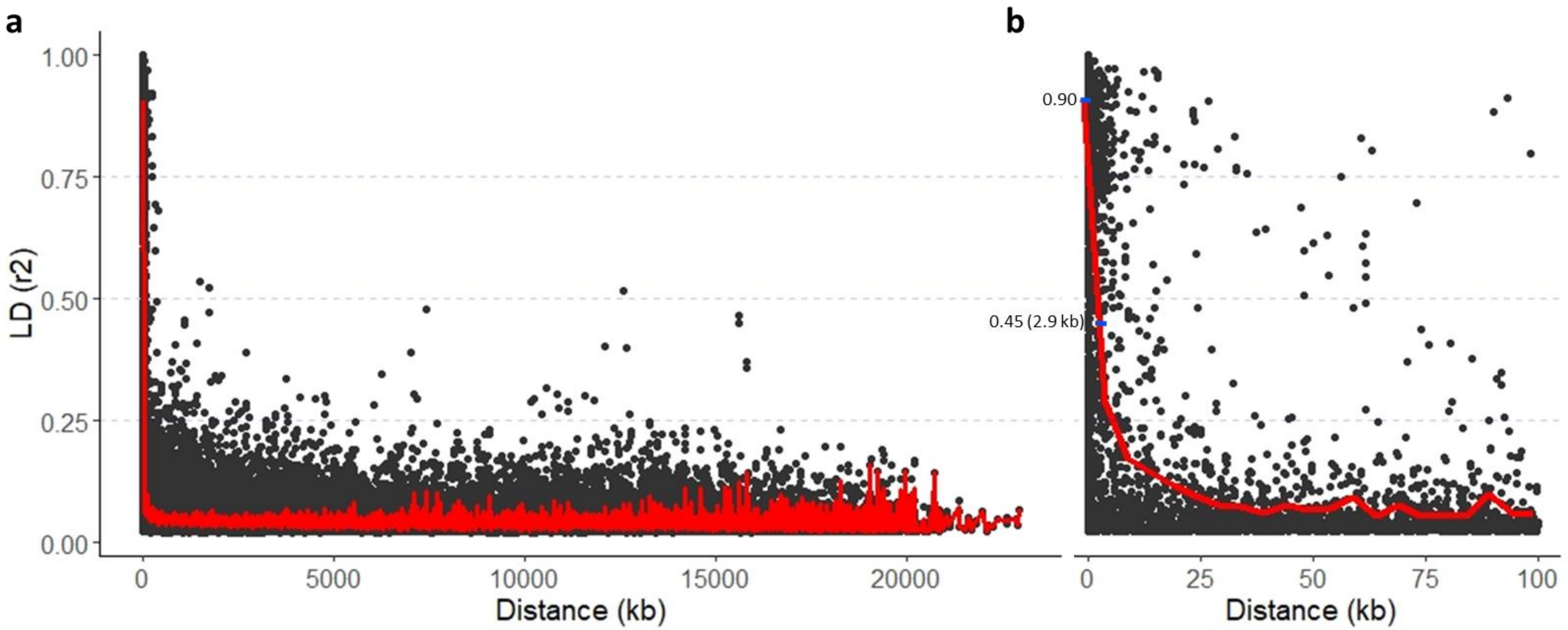

ESM_6: Genome-wide linkage disequilibrium (LD) decay based on 181 white lupin accessions. a: Whole genome ~23000 kb. **b:** 100 kb, the decay of LD with physical distance between SNPs to half of the maximum values occurred at 2.9 kb (r^2 =0.45).

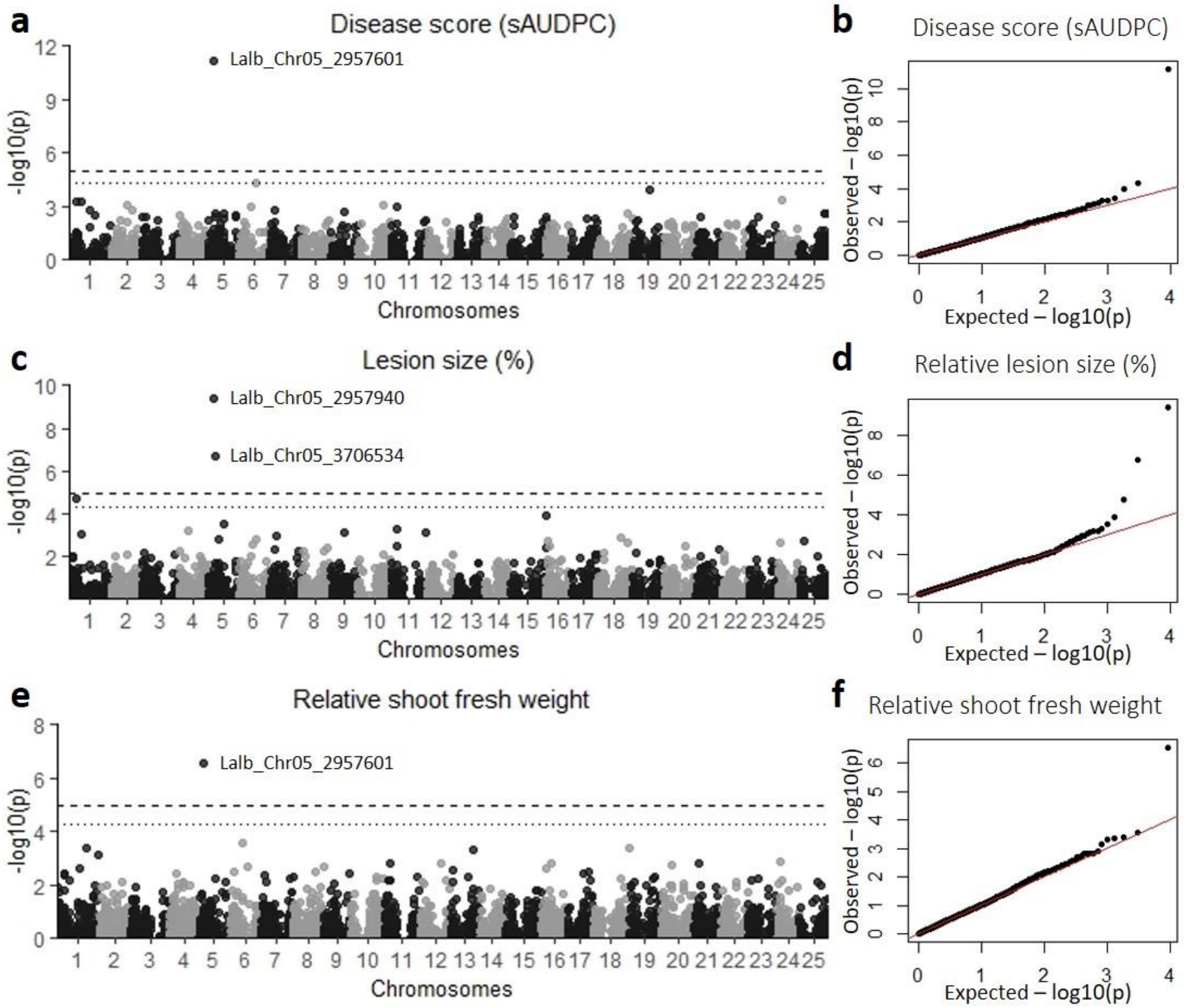

ESM_7: Manhattan and corresponding Q-Q plots by BLINK showing SNP association with anthracnose resistance. a-b: Disease score (standardized area under the disease progress curve (sAUDPC)). **c-d:** Relative lesion size (%). **e-f:** Relative shoot fresh weight. Upper dashed line indicates Bonferroni corrected LOD threshold of 4.96 (*P* = 1.08E-05) and lower dotted line indicates fixed LOD threshold of 4.3 (*P* = 5.00E-05).

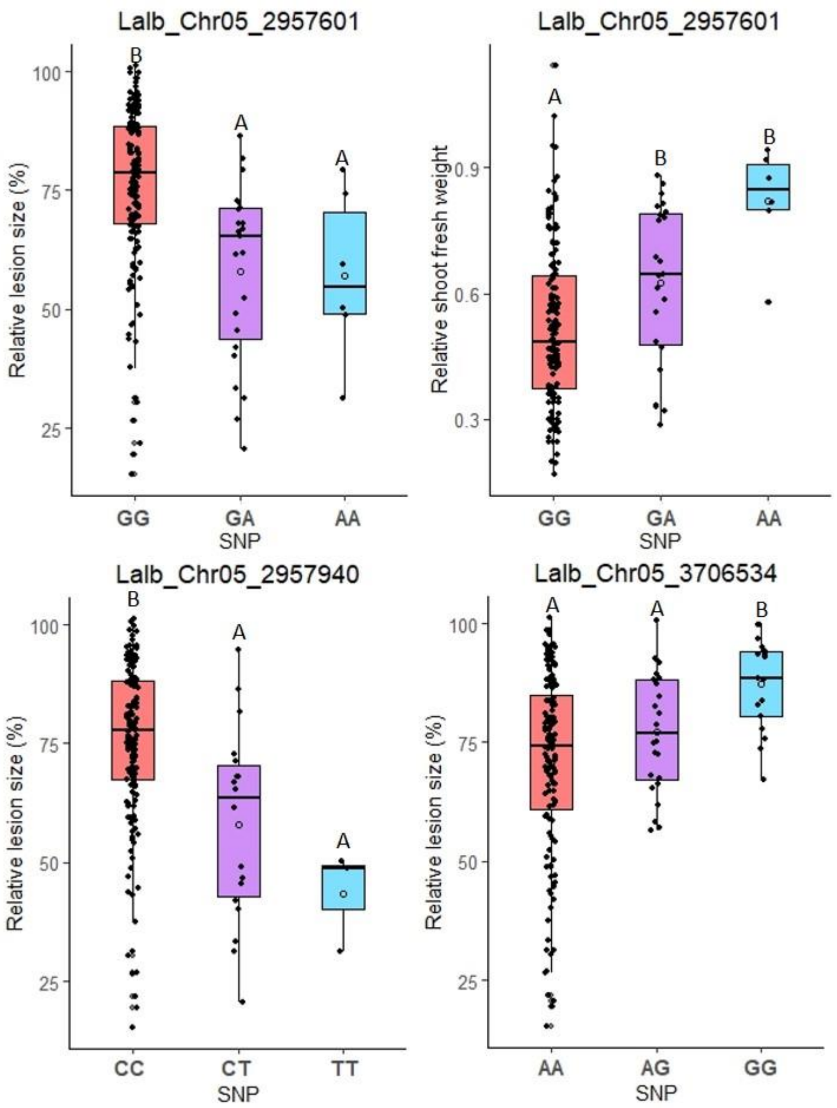

ESM_8: Boxplots showing allele effect on relative lesion size (%) and relative shoot fresh weight for SNPs associated with anthracnose resistance. Capital letters within plot indicate significant difference (Tukey-HSD, *P* ≤ 0.05). Bonferroni LOD threshold of 4.96 (P = 1.08E-05) was used to identify significant SNPs.

ESM_9: SNPs above fixed threshold and associated linked SNPs and candidate genes [a].

Trait	SNP[b]	Allele	P value MLM	P value BLINK	R^2_{LR}	MAF	Candidate gene(s)	Location[c]	Annotation
Disease Score (sAUDPC)	Lalb_Chr06_9655085	A/G	5.56E-04	4.76E-05	0.06	0.07	*Lalb_Chr06g0172191*	Intron	PPC domain-containing protein
Relative Lesion size (%)	Lalb_Chr01_3872625	A/T	7.40E-04	1.83E-05	0.06	0.32	*Lalb_Chr01g0006951*	Intron	Glucan endo-1,3-beta-D-glucosidase
							Lalb_Chr01g0006961	2.5 kb >	Winged helix-turn-helix DNA-binding domain, leucine-rich repeat domain
							Lalb_Chr01g0006941	2 kb <	Putative chromatin regulator PHD family
	Lalb_Chr05_3688076[d]	A/G	0.52	0.54	0.00	0.16	*Lalb_Chr05 g0217331*	Exon	RNA helicase
	Lalb_Chr05_3784474[d]	A/G	0.68	0.87	0.00	0.15	*Lalb_Chr05 g0217471*	Exon	LRR receptor serine/threonine-protein kinase
Relative shoot fresh weight	Lalb_Chr13_12108967	G/A	2.52E-05	4.79E-04	0.09	0.43	*Lalb_Chr13g0297911*	Exon	Protease Do-like 9
							Lalb_Chr13g0297901	3 kb <	RING-H2 finger protein
	Lalb_Chr13_12143224	C/A	1.22E-02	1.41E-02	0.03	0.09	*Lalb_Chr13g0297971*	Intron	Betaine aldehyde dehydrogenase

[a] SNP = Single-nucleotide polymorphism, MAF = minor allele frequency, R^2_{LR} = likelihood-ratio-based R^2, sAUDPC = standardized area under the disease progress curve, fixed LOD threshold is 4.3 (P = 5.00E-05). [b] Lalb = *Lupinus albus*, Chr = chromosome, number = position on chromosome. [c] < = downstream, > = upstream. [d] Linked to SNP Lalb_Chr05_3706534, see **Table 1**.
